# Relationship between telomere length and the prognosis of breast cancer based on estrogen receptor status: A Mendelian randomization study

**DOI:** 10.3389/fonc.2022.1024772

**Published:** 2022-10-21

**Authors:** Yilun Li, Li Ma

**Affiliations:** The Fourth Hospital of Hebei Medical University, Shijiazhuang, Hebei, China

**Keywords:** telomere length, breast cancer, estrogen receptor (ER), ER status, Mendelian randomization study

## Abstract

**Objective:**

To identify the relationship between telomere length and the prognosis of breast cancer with different status of estrogen receptor (ER).

**Methods:**

We collected single nucleotide polymorphisms (SNPs) associated with telomere length and breast cancer prognosis from the MRCIEU GWAS database and the dataset of a large meta-analysis conducted by the Breast Cancer Association Consortium (BCAC), respectively. The relationship was identified using inverse-variance weighted (IVW), MR-Egger, weighted median, penalized weighted median, and maximum likelihood methods. IVW, MR-Egger, and MR-PRESSO methods were used to perform sensitivity analysis to assess the accuracy of the results.

**Results:**

Telomere length was negatively associated with the prognosis of total breast cancer (odds ratio [OR]=1.84, 95% confidence interval [CI]=1.08-3.14, IVW method), especially with ER- breast cancer (OR=1.89, 95% CI=1.11-3.22, IVW method). No similar relationship was found between telomere length and the prognosis of ER+ breast cancer (OR=0.99, 95% CI=0.62-1.58, IVW method). The findings from other methods were consistent with the results shown by the IVW method. The Mendelian randomization assumptions did not appear to be violated. Sensitivity analysis indicated that the result was robust, and no bias was observed in the study.

**Conclusion:**

Telomere length is associated with the prognosis of total breast cancer, especially with ER- breast cancer. There is no significant correlation between telomere length and the prognosis of ER+ breast cancer. These findings add to the evidence that long telomere could predict a poor prognosis of ER- breast cancer.

## Introduction

Breast cancer is one of the most common cancers in women worldwide. An estimated 287,850 American women were diagnosed with breast cancer in 2022, resulting in 61,360 deaths (15% of women’s cancer mortality) (1, 2).

Several factors affect the risk and mortality rate of breast cancer, such as first-degree family history of breast cancer, early age at menarche, late age at first birth, late age at menopause, overweight or obesity, use of oral contraceptive, and exogenous hormone (3). These factors account for 70% of postmenopausal women with breast cancer in the USA (4, 5). The high incidence and mortality rate of breast cancer threaten women’s physical and mental health. Therefore, more predictors are required to identify patients with breast cancer and help doctors formulate personalized breast cancer treatment plans.

The estrogen receptor (ER) plays an important role in breast cancer. About 70% of breast cancer cases could be detected in the expression of ER (6). Its biological characteristics and prognosis are distinctly different from other subtypes, which show sensitivity to anti-hormone therapy (7). Compared to patients with estrogen receptor-negative (ER-, ER<1% is considered ER-) breast cancer, patients with estrogen receptor-positive (ER+, ER>=1% is considered ER+) breast cancer had a better prognosis (8). European Society for Medical Oncology (ESMO) and National Comprehensive Cancer Network (NCCN) guidelines also include ER as an important prognostic indicator for breast cancer (9, 10).

The telomere is a tandem repeat sequence of TTAGGG located at the distal end of the linear chromosome (11, 12). It plays a vital role in maintaining structural integrity and regulating cell replication by preventing DNA double-strand breaks, end-to-end chromosome fusion, and degradation (13). Telomeres shorten with the cell division cycle and are generally considered a marker of aging at the cellular level in organisms (14). Thus, telomeres have been extensively studied as biomarkers for aging and age-related diseases, such as cardiovascular diseases, cancer, and diabetes (15).

The relationship between telomere length and the incidence and prognosis of breast cancer is still unclear. Several studies have shown a positive relationship between telomere length and the risk of breast cancer (16–18), some have reported a negative correlation (19, 20), while other studies show a null association (21–23). Regarding the prognosis of breast cancer, one study shows that telomere length is negatively correlated with breast cancer prognosis (24), while another study reports a positive correlation (25). Several studies did not find any association between breast cancer prognosis and telomere length (26, 27). Furthermore, only a few studies have investigated the relationship between telomere length and the incidence of breast cancer based on the status of ER (28, 29). There is a lack of studies on the relationship between telomere length and the prognosis of breast cancer with different status of ER.

These inconsistent findings mentioned above can be attributed to several confounding factors. Due to inherent flaws in traditional designs, existing observational studies cannot completely rule out possible factors of reverse causation and confounding, leading to biased associations and conclusions (30). Mendelian randomization (MR) is one approach that can address these limitations (31). MR applies genetic variations associated with environmental exposures as instrumental variables (IVs) to assess associated exposures (e.g., telomere length) and outcomes (e.g., the prognosis of breast cancer with different status of ER) (32). Since alleles are randomly assigned at conception according to Mendel’s second law (33), MR analysis can effectively eliminate the effect of confounding factors and identify causal determinants of a certain outcome.

This study aimed to identify the causal association between telomere length and the prognosis of breast cancer with different status of ER. To this end, we used two-sample MR to analyze the effect of telomere length on the prognosis of total breast cancer. Next, we individually evaluated the relationship between telomere length and the prognosis of ER+ and ER- breast cancer.

## Material and methods

### Data collection

We collected single nucleotide polymorphisms (SNPs) related to exposure and outcome. SNPs associated with telomere length (exposure) were obtained from the MRCIEU GWAS database (https://gwas.mrcieu.ac.uk/). The database includes 472,174 samples, containing 20,134,421 SNPs in the exposure dataset. SNPs related to breast cancer survival with different status of ER were collected from the dataset of a large meta-analysis conducted by the Breast Cancer Association Consortium (BCAC) (34), which included 37,954 samples and 12,940,150 SNPs. Of these, 6,881 samples and 8,828,662 SNPs related to breast cancer survival with ER- status, and 23,059 samples and 8,714,606 SNPs associated with breast cancer survival with ER+ status. All data belonged to the population of Europe. The original data are presented as **Supplementary Material** (**Tables S1**–**S3**).

### Instrumental variable extraction

SNPs were selected as IVs to evaluate the causal effects of telemore length on the risk of breast cancer in accordance with the following assumptions (1): genetic variants must be strongly associated with exposure (*P*<5×10^-8^); (2) genetic variants cannot be associated with any potential confounders; (3) genetic variants affect the outcome only *via* the risk factors (35). The window of linkage disequilibrium (LD) was set r^2^<0.01 at 10,000 kb to ensure the independence of the selected genetic variation. These SNPs were examined for the potential violations of assumptions (2) and (3) based on the PhenoScanner database (http://www.phenoscanner.medschl.cam.ac.uk/) (36); SNPs closely related to breast cancer survival were excluded (BMI, weight, smoking, cholesterol) (37–39). We also examined the possible pleiotropy of the selected SNPs using the MR Pleiotropy RESidual Sum and Outlier (MR-PRESSO) test, and no SNPs were excluded. Besides, palindromic SNPs with intermediate allele frequencies were also removed. Furthermore, all data were extracted from the European population, which could decrease the influence of population stratification. According to the above inclusion and exclusion criteria, we excluded inappropriate IVs. Besides, multiple methods were used in the study to ensure the accuracy of the results.

Finally, 104 SNPs (total breast cancer survival), 99 SNPs (breast cancer survival with ER+ status), and 100 SNPs (breast cancer survival with ER- status) were included for further study.

### Mendelian randomization analysis

Inverse-variance weighted (IVW) method was used for preliminary analysis to assess the causal relationship between telomere length and the prognosis of breast cancer with different status of ER. Inverse variance weighting is a method of aggregating two or more random variables to minimize the variance of the sum, the weighting of each random variable in the sum is inversely proportional to its variance, which is often used to combine results from independent studies (35). The exposure-outcome effect for each SNP was calculated using the Wald ratio method. To ensure the accuracy of results across a wider range of scenarios, multiple methods including MR-Egger regression, weighted median, penalized weighted median, and maximum likelihood were also performed.

### Sensitivity analysis

IVW and MR-Egger methods were applied in the leave-one-out analysis to evaluate the combined effect of the remaining SNPs. If the combined effect was consistent with the main effect, this indicated that no single SNP had an excessive influence on MR analysis. Funnel plot and MR-Egger intercept tests were also performed to detect the presence of pleiotropy and assess the robustness of the results. Heterogeneity was evaluated by IVW and MR-Egger tests; *P* value <0.05 indicated the presence of heterogeneity in the study. MR-PRESSO R package was used to assess whether or not there was any difference between the results of MR analysis before and after correction (40).

### Statistical analysis

The results of MR estimates were shown as odds ratios (ORs) with corresponding 95% confidence intervals (CIs). We applied R software (Version 4.1.2), using the R package (TwoSampleMR, MR-PRESSO) to perform MR analysis and sensitivity analysis; R package “forestplot” was used to plot figures. A two-sided *P* value <0.05 was considered statistically significant.

## Results

### Mendelian randomization

**Figure 1:**
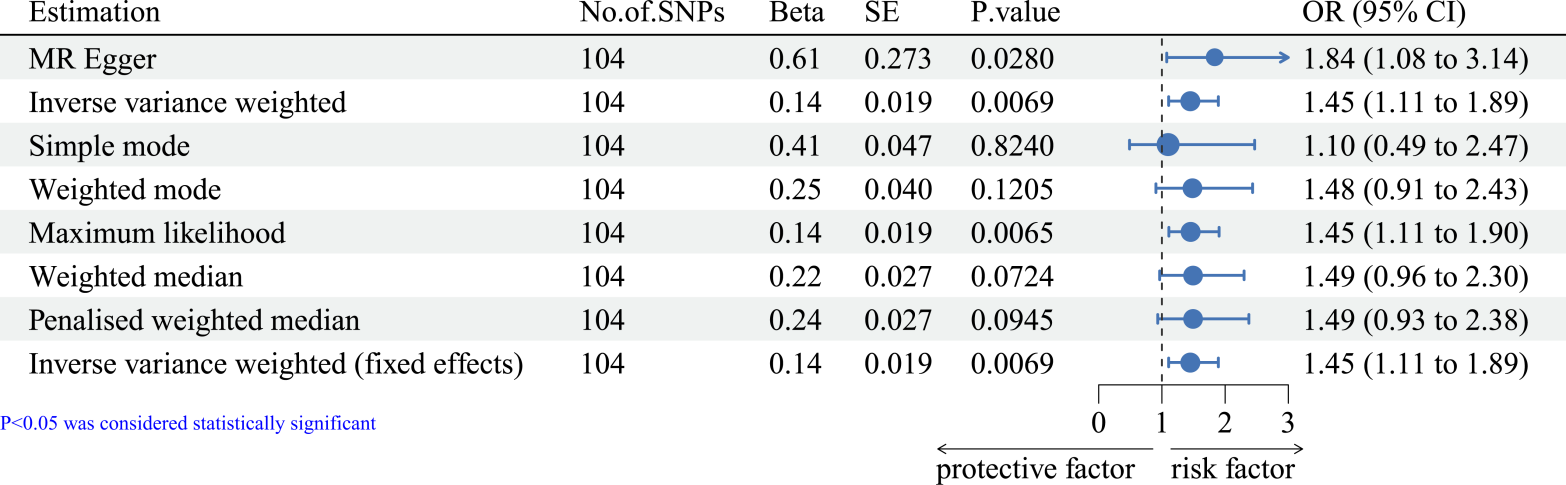
Forest plot of MR methods of the effect of telomere length on the prognosis of total breast cancer. MR, Mendelian randomization.

MR analysis showed that telomere length was negatively associated with the prognosis of total breast cancer (see **Figure 1**, OR=1.84, 95% CI=1.08-3.14, IVW method), indicating that telomere length is a risk factor in breast cancer prognosis.

**Figure 2:**
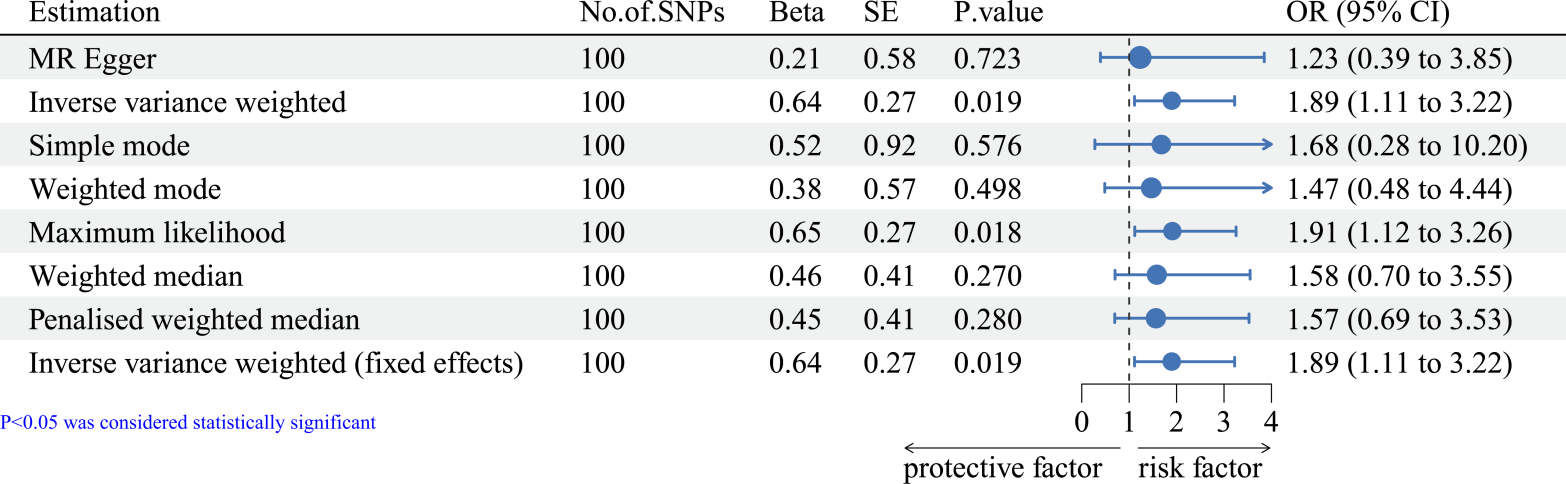
Forest plot of MR methods of the effect of telomere length on the prognosis of ER- breast cancer. MR, Mendelian randomization.

**Figure 3:**
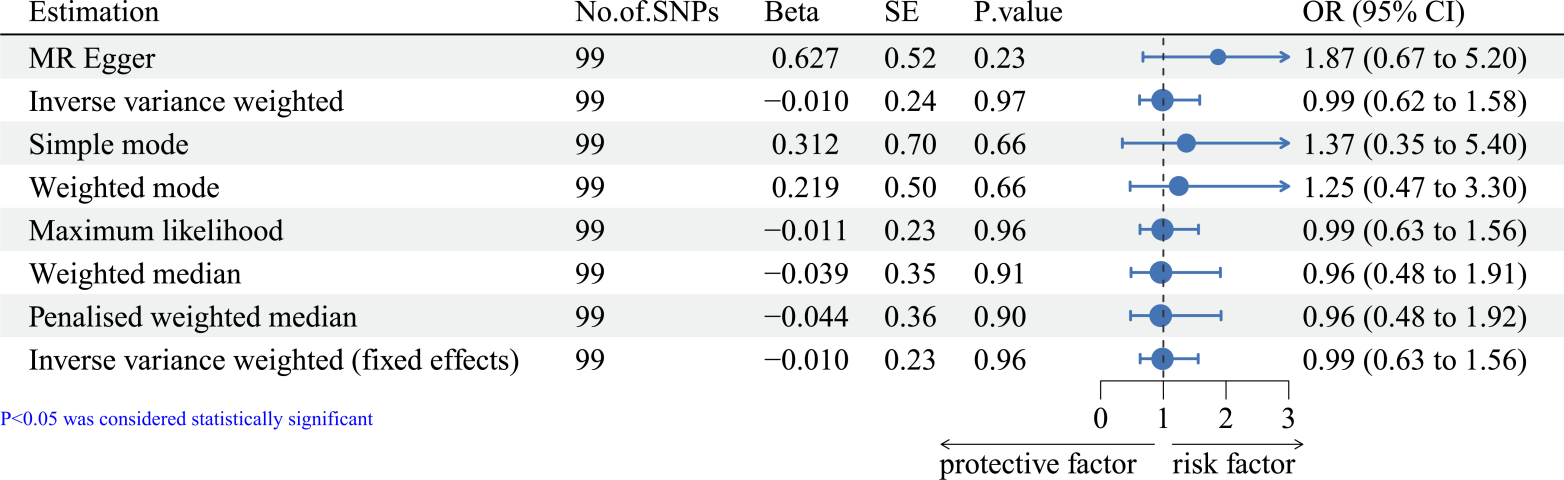
Forest plot of MR methods of the effect of telomere length on the prognosis of ER+ breast cancer. MR, Mendelian randomization.

Telomere length was also negatively associated with the prognosis of ER- breast cancer (see **Figure 2**, OR=1.89, 95% CI=1.11-3.22, IVW method), suggesting that telomere length was a risk factor in the prognosis of breast cancer with ER- status. Interestingly, no similar relationship was found between telomere length and the prognosis of breast cancer with ER+ status (see **Figure 3**, OR=0.99, 95% CI=0.62-1.58, IVW method).

To ensure the accuracy of the findings, we also evaluated the correlations using other methods, the results of which were consistent considering the prognosis of breast cancer with different ER status (see **Figures 1**–**3**).

### Sensitivity analysis

We also performed a sensitivity analysis to ensure the accuracy of the results. There was no heterogeneity in the IVW test (Q=100.710, *P*=0.545) and MR-Egger test (Q=99.691, *P*=0.545) (**Table 1**) with regard to total breast cancer. No significant heterogeneity was observed in both ER- and ER+ breast cancer (**Table 1**). MR-Egger intercept test showed *P* value >0.05, suggesting the non-existence of horizontal pleiotropy (**Table 1**). MR-PRESSO test ensured the accuracy of the results (**Table 1**). Furthermore, no single SNP showed a significant impact on the MR estimation results based on the leave-one-out analysis (**Supplementary Figure S1**). None of the estimates were violated based on the funnel plots (**Supplementary Figure S2**).

**Table 1:** Sensitivity analysis of the causal association between telomere length and the prognosis of breast cancer with different status of estrogen receptor (ER).

ER status	Heterogeneity				Pleiotropy		Outlier examination by MR-PRESSO					
	MR-Egger		Inverse variance weighted		MR-Egger		Before correction			After correction (if necessary)		
	Q	*P* value	Q	*P* value	Intercept	*P* value	MR Analysis Causal Estimate	SD	*P* value	MR Analysis Causal Estimate	SD	*P* value
ER+	102.709	0.326	104.708	0.303	-0.017	0.173	0.021	0.237	0.930	NA	NA	NA
ER-	90.291	0.698	90.994	0.704	0.011	0.404	0.639	0.260	0.016	NA	NA	NA
Total	99.691	0.546	100.710	0.545	-0.007	0.315	0.370	0.135	0.007	NA	NA	NA

## Discussion

The study results show that telomere length is negatively associated with the prognosis of breast cancer, especially in ER- breast cancer, while there is no significant relationship between telomere length and the prognosis of ER+ breast cancer.

As mentioned in the Introduction, research shows that telomere length is negatively correlated with the incidence of breast cancer (19, 20). However, a meta-analysis of prospective studies including approximately 14,000 cases has shown that longer leukocyte telomere length was marginally associated with an increased risk of total breast cancer incidence (41). Another study also found a positive association between longer telomere length and increased risk of breast cancer (18). The possible mechanism is that blood lymphocytes may be stimulated during inflammation and tumorigenesis and regulate telomerase through the NF-κB pathway, thereby regulating telomere length (42). Long telomeres may allow damaged cells to survive longer and continue to divide and acquire additional mutations, resulting in malignant transformation (43).

Telomere length is also associated with the prognosis of breast cancer. Research shows that long telomere predicts a good prognosis in breast cancer (25). However, another study found a negative correlation between telomere length and the prognosis of breast cancer (24). It could be explained by the mechanism that maintaining telomere length is required for the continuous growth of the tumor, especially in advanced tumor (44). Cancer cells can maintain their immortality by reactivating or up-regulating telomerase, another possible mechanism is that cancer cells can reverse telomere attrition in order to bypass senescence that is termed the alternative lengthening of telomeres pathway that involves DNA recombination between telomeres to achieve the immortality (45). Our results are consistent with the findings of this study. The genetic predisposition to long telomeres may influence cancer mortality through the telomere maintenance pathway (18). One explanation is that the rate of telomere shortening in breast cancer cells is slowed, and apoptosis is reduced when the immune system is suppressed (46). Immune suppression is found to be associated with a bad prognosis of breast cancer (18). Another explanation is that cells with very short telomeres may induce replicative senescence or apoptosis, thereby inhibiting the proliferative potential of the cells and thus supporting tumor suppressor activity (11, 47). The specific functional mechanisms of telomeres in cancer are still unclear. Further studies are needed to identify these mechanisms.

Hormones are also strongly associated with telomere length. The present study shows that long telomere length is related to a poor prognosis of ER- breast cancer. A study evaluating long telomere length of ER expression in 200 breast cancer patients did not find any statistically significant difference in the prognosis between ER+ and ER- patients, but it did find that ER+ cases had longer telomere length compared to control cases (16). This is because estrogen is directly involved in telomerase activation promotion through its action on the effects of human telomerase reverse transcriptase (hTERT) and post-transcriptional modification by AKT-dependent phosphorylation of hTERT (48). However, another study did not find any significant correlation between telomere length and breast cancer with different ER status (26). Further research is required to clarify the specific mechanism of estrogen action on telomeres.

The present study has several limitations. First, because of a lack of secondary data, we were unable to conduct a stratified analysis on progesterone receptor (PR) and human epidermal growth factor receptor 2 (HER2). Second, the present study had a small sample size; future studies should include a bigger sample population to improve the universality of the conclusion. Third, this study included data from only the European population. Future research should focus on other population samples.

## Conclusion

This study shows that telomere length is associated with the prognosis of breast cancer, especially in ER- breast cancer; however, there is no significant correlation between telomere length and the prognosis of ER+ breast cancer. These findings suggest that long telomere could predict a poor prognosis of ER- breast cancer.

## Data availability statement

The original contributions presented in the study are included in the article/**Supplementary Material**. Further inquiries can be directed to the corresponding author.

## Author contributions

Conceptualization, LM; methodology, YL; software, YL; validation, LM and YL; formal analysis, YL; investigation, LM; resources, YL; data curation, YL; writing—original draft preparation, YL; writing—review and editing, YL; visualization, YL; supervision, LM; project administration, LM. All authors have read and agreed to the published version of the manuscript.

## Conflict of interest

The authors declare that the research was conducted in the absence of any commercial or financial relationships that could be construed as a potential conflict of interest.

## Publisher’s note

All claims expressed in this article are solely those of the authors and do not necessarily represent those of their affiliated organizations, or those of the publisher, the editors and the reviewers. Any product that may be evaluated in this article, or claim that may be made by its manufacturer, is not guaranteed or endorsed by the publisher.

## Supplementary material

Supplementary Table 1 Characteristics of SNPs associated with telomere length and the prognosis of total breast cancer.

Supplementary Table 2 Characteristics of SNPs associated with telomere length and the prognosis of ER- breast cancer.

Supplementary Table 3 Characteristics of SNPs associated with telomere length and the prognosis of ER+ breast cancer.

Supplementary Figure 1 Sensitivity analysis based on leave-one-out analysis.

Supplementary Figure 2 Funnel plot to assess the robustness of results. Scattering points represent the effect estimated using a single SNP as an instrumental variable. Vertical lines denote the overall estimate obtained by the inverse variance weighted estimate and the MR-Egger regression.

## Abbreviations

ER, Estrogen receptor; ER-, Estrogen receptor-negative; ER+, Estrogen receptor-positive; MR, Mendelian randomization; IVs, Instrumental variables; SNP, Single nucleotide polymorphisms; BCAC, Breast Cancer Association Consortium; BMI, Body mass index; LD, Linkage disequilibrium; IVW, Inverse-variance weighted; OR, Odds ratio; CI, Confidence interval; PR, progesterone receptor; HER2, Human epidermal growth factor receptor 2.

## References

[1] Giaquinto AN Sung H Miller KD Kramer JL Newman LA Minihan A. Breast cancer statistics, 2022. CA: Cancer J Clin (2022) 0:1–18. doi: 10.3322/caac.21754 36190501

[2] Siegel RL Miller KD Fuchs HE Jemal A. Cancer statistics, 2022. CA: Cancer J Clin (2022) 72(1):7–33. doi: 10.3322/caac.21708 35020204

[3] Mina L Storniolo AM Kipfer HD Hunter C Ludwig K. Breast cancer prevention and treatment. Breast Cancer Risk Factors (2016) (Chapter2):5–11. doi: 10.1007/978-3-319-19437-0

[4] Tamimi RM Spiegelman D Smith-Warner SA Wang M Pazaris M Willett WC. Population attributable risk of modifiable and nonmodifiable breast cancer risk factors in postmenopausal breast cancer. Am J Epidemiol (2016) 184(12):884–93. doi: 10.1093/aje/kww145 PMC5161087 27923781

[5] Engmann NJ Golmakani MK Miglioretti DL Sprague BL Kerlikowske K. Population-attributable risk proportion of clinical risk factors for breast cancer. JAMA Oncol (2017) 3(9):1228–36. doi: 10.1001/jamaoncol.2016.6326 PMC5540816 28152151

[6] Howlader N Altekruse SF Li CI Chen VW Clarke CA Ries LA. US Incidence of breast cancer subtypes defined by joint hormone receptor and HER2 status. J Natl Cancer Inst (2014) 106(5). doi: 10.1093/jnci/dju055 PMC4580552 24777111

[7] Zhu Y Wang J Xu B. A novel prognostic nomogram for predicting survival of hormone receptor-positive and HER2 negative advanced breast cancer among the han-population. Front Oncol (2022) 12:918759. doi: 10.3389/fonc.2022.918759 35847941 PMC9285102

[8] Stenmark Tullberg A Lundstedt D Olofsson Bagge R Karlsson P. Positive sentinel node in luminal a-like breast cancer patients - implications for adjuvant chemotherapy? Acta Oncol (Stockholm Sweden) (2019) 58(2):162–7. doi: 10.1080/0284186X.2018.1533647 30407093

[9] Cardoso F Paluch-Shimon S Senkus E Curigliano G Aapro MS André F. 5th ESO-ESMO international consensus guidelines for advanced breast cancer (ABC 5). Ann Oncol Off J Eur Soc Med Oncol (2020) 31(12):1623–49. doi: 10.1016/j.annonc.2020.09.010 PMC7510449 32979513

[10] Gradishar WJ Anderson BO Abraham J Aft R Agnese D Allison KH. Breast cancer, version 3.2020, NCCN clinical practice guidelines in oncology. J Natl Compr Cancer Netw JNCCN (2020) 18(4):452–78. doi: 10.6004/jnccn.2020.0016 32259783

[11] Blasco MA. Telomeres and human disease: ageing, cancer and beyond. Nat Rev Genet (2005) 6(8):611–22. doi: 10.1038/nrg1656 16136653

[12] Blackburn EH. Telomeres and telomerase: their mechanisms of action and the effects of altering their functions. FEBS Letters (2005) 579(4):859–62. doi: 10.1016/j.febslet.2004.11.036 15680963

[13] Aubert G Lansdorp PM. Telomeres and aging. Physiol Rev (2008) 88(2):557–79. doi: 10.1152/physrev.00026.2007 18391173

[14] Samavat H Luu HN Beckman KB Jin A Wang R Koh WP. Leukocyte telomere length, cancer incidence and all-cause mortality among Chinese adults: Singapore Chinese health study. Int J Cancer (2021) 148(2):352–62. doi: 10.1002/ijc.33211 PMC10693991 33459354

[15] Sanders JL Newman AB. Telomere length in epidemiology: A biomarker of aging, age-related disease, both, or neither? Epidemiologic Rev (2013) 35(1):112–31. doi: 10.1093/epirev/mxs008 PMC4707879 23302541

[16] Svenson U Nordfjäll K Stegmayr B Manjer J Nilsson P Tavelin B. Breast cancer survival is associated with telomere length in peripheral blood cells. Cancer Res (2008) 68(10):3618–23. doi: 10.1158/0008-5472.CAN-07-6497 18483243

[17] Gramatges MM Telli ML Balise R Ford JM. Longer relative telomere length in blood from women with sporadic and familial breast cancer compared with healthy controls. Cancer Epidemiology Biomarkers Prev Publ Am Assoc Cancer Research cosponsored by Am Soc Prev Oncol (2010) 19(2):605–13. doi: 10.1158/1055-9965.EPI-09-0896 20142254

[18] Samavat H Xun X Jin A Wang R Koh WP Yuan JM. Association between prediagnostic leukocyte telomere length and breast cancer risk: The Singapore Chinese health study. Breast Cancer Res BCR (2019) 21(1):50. doi: 10.1186/s13058-019-1133-0 30995937 PMC6471852

[19] Pooley KA Sandhu MS Tyrer J Shah M Driver KE Luben RN. Telomere length in prospective and retrospective cancer case-control studies. Cancer Res (2010) 70(8):3170–6. doi: 10.1158/0008-5472.CAN-09-4595 PMC2855947 20395204

[20] Qu S Wen W Shu XO Chow WH Xiang YB Wu J. Association of leukocyte telomere length with breast cancer risk: Nested case-control findings from the shanghai women's health study. Am J Epidemiol (2013) 177(7):617–24. doi: 10.1093/aje/kws291 PMC3657533 23444102

[21] Kim S Sandler DP Carswell G De Roo LA Parks CG Cawthon R. Telomere length in peripheral blood and breast cancer risk in a prospective case-cohort analysis: results from the sister study. Cancer Causes Control CCC (2011) 22(7):1061–6. doi: 10.1007/s10552-011-9778-8 PMC3445257 21643930

[22] Rode L Nordestgaard BG Bojesen SE. Long telomeres and cancer risk among 95 568 individuals from the general population. Int J Epidemiol (2016) 45(5):1634–43. doi: 10.1093/ije/dyw179 27498151

[23] De Vivo I Prescott J Wong JY Kraft P Hankinson SE Hunter DJ. A prospective study of relative telomere length and postmenopausal breast cancer risk. Cancer epidemiology Biomarkers Prev Publ Am Assoc Cancer Research cosponsored by Am Soc Prev Oncol (2009) 18(4):1152–6. doi: 10.1158/1055-9965.EPI-08-0998 PMC2732000 19293310

[24] Thriveni K Raju A Kumar RV Krishnamurthy S Chaluvarayaswamy R. Patterns of relative telomere length is associated with hTERT gene expression in the tissue of patients with breast cancer. Clin Breast Cancer (2019) 19(1):27–34. doi: 10.1016/j.clbc.2018.07.021 30217473

[25] Gay-Bellile M Romero P Cayre A Véronèse L Privat M Singh S. ERCC1 and telomere status in breast tumours treated with neoadjuvant chemotherapy and their association with patient prognosis. J Pathol Clin Res (2016) 2(4):234–46. doi: 10.1002/cjp2.52 PMC5068194 27785368

[26] Kroupa M Rachakonda S Vymetalkova V Tomasova K Liska V Vodenkova S. Telomere length in peripheral blood lymphocytes related to genetic variation in telomerase, prognosis and clinicopathological features in breast cancer patients. Mutagenesis (2020) 35(6):491–7. doi: 10.1093/mutage/geaa030 33367858

[27] Vodenkova S Kroupa M Polivkova Z Musak L Ambrus M Schneiderova M. Chromosomal damage and telomere length in peripheral blood lymphocytes of cancer patients. Oncol Rep (2020) 44(5):2219–30. doi: 10.3892/or.2020.7774 33000239

[28] Chen F Wen W Long J Shu X Yang Y Shu XO. Mendelian randomization analyses of 23 known and suspected risk factors and biomarkers for breast cancer overall and by molecular subtypes. Int J Cancer (2022) 151(3):372–80. doi: 10.1002/ijc.34026 PMC9177773 35403707

[29] Carugno M Maggioni C Crespi E Bonzini M Cuocina S Dioni L. Night shift work, DNA methylation and telomere length: An investigation on hospital female nurses. Int J Environ Res Public Health (2019) 16(13):2292. doi: 10.3390/ijerph16132292 PMC6651131 31261650

[30] Sekula P Del Greco MF Pattaro C Köttgen A. Mendelian randomization as an approach to assess causality using observational data. J Am Soc Nephrol JASN (2016) 27(11):3253–65. doi: 10.1681/ASN.2016010098 PMC5084898 27486138

[31] Davey Smith G Hemani G. Mendelian randomization: Genetic anchors for causal inference in epidemiological studies. Hum Mol Genet (2014) 23(R1):R89–98. doi: 10.1093/hmg/ddu328 PMC4170722 25064373

[32] Burgess S Thompson SG. Multivariable mendelian randomization: the use of pleiotropic genetic variants to estimate causal effects. Am J Epidemiol (2015) 181(4):251–60. doi: 10.1093/aje/kwu283 PMC4325677 25632051

[33] Smith GD Ebrahim S. 'Mendelian randomization': Can genetic epidemiology contribute to understanding environmental determinants of disease? Int J Epidemiol (2003) 32(1):1–22. doi: 10.1093/ije/dyg070 12689998

[34] Guo Q Schmidt MK Kraft P Canisius S Chen C Khan S. Identification of novel genetic markers of breast cancer survival. J Natl Cancer Inst (2015) 107(5). doi: 10.1093/jnci/djv081 PMC4555642 25890600

[35] Ma M Zhi H Yang S Yu EY Wang L. Body mass index and the risk of atrial fibrillation: A mendelian randomization study. Nutrients (2022) 14(9):1878. doi: 10.3390/nu14091878 35565843 PMC9101688

[36] Staley JR Blackshaw J Kamat MA Ellis S Surendran P Sun BB. PhenoScanner: a database of human genotype-phenotype associations. Bioinf (Oxford England) (2016) 32(20):3207–9. doi: 10.1093/bioinformatics/btw373 PMC5048068 27318201

[37] Jones ME Schoemaker MJ Wright LB Ashworth A Swerdlow AJ. Smoking and risk of breast cancer in the generations study cohort. Breast Cancer Res BCR (2017) 19(1):118. doi: 10.1186/s13058-017-0908-4 29162146 PMC5698948

[38] Garcia-Estevez L Moreno-Bueno G. Updating the role of obesity and cholesterol in breast cancer. Breast Cancer Res BCR (2019) 21(1):35. doi: 10.1186/s13058-019-1124-1 30823902 PMC6397485

[39] Patrício M Pereira J Crisóstomo J Matafome P Gomes M Seiça R. Using resistin, glucose, age and BMI to predict the presence of breast cancer. BMC Cancer (2018) 18(1):29. doi: 10.1186/s12885-017-3877-1 29301500 PMC5755302

[40] Verbanck M Chen CY Neale B Do R. Detection of widespread horizontal pleiotropy in causal relationships inferred from mendelian randomization between complex traits and diseases. Nat Genet (2018) 50(5):693–8. doi: 10.1038/s41588-018-0099-7 PMC6083837 29686387

[41] Zhang X Zhao Q Zhu W Liu T Xie SH Zhong LX. The association of telomere length in peripheral blood cells with cancer risk: A systematic review and meta-analysis of prospective studies. Cancer Epidemiology Biomarkers Prev Publ Am Assoc Cancer Research cosponsored by Am Soc Prev Oncol (2017) 26(9):1381–90. doi: 10.1158/1055-9965.EPI-16-0968 28619828

[42] Ghosh A Saginc G Leow SC Khattar E Shin EM Yan TD. Telomerase directly regulates NF-κB-dependent transcription. Nat Cell Biol (2012) 14(12):1270–81. doi: 10.1038/ncb2621 23159929

[43] Aviv A Anderson JJ Shay JW. Mutations, cancer and the telomere length paradox. Trends Cancer (2017) 3(4):253–8. doi: 10.1016/j.trecan.2017.02.005 PMC5903276 28718437

[44] Tabori U Vukovic B Zielenska M Hawkins C Braude I Rutka J. The role of telomere maintenance in the spontaneous growth arrest of pediatric low-grade gliomas. Neoplasia (New York NY) (2006) 8(2):136–42. doi: 10.1593/neo.05715 PMC1578515 16611406

[45] Shay JW. Role of telomeres and telomerase in aging and cancer. Cancer Discov (2016) 6(6):584–93. doi: 10.1158/2159-8290.CD-16-0062 PMC4893918 27029895

[46] Hanahan D Weinberg RA. Hallmarks of cancer: the next generation. Cell (2011) 144(5):646–74. doi: 10.1016/j.cell.2011.02.013 21376230

[47] Li S Xu W Harley CB. Telomere loss: mitotic clock or genetic time bomb? World J Gastroenterol (1934). 10.1016/0921-8734(91)90018-7 1722017

[48] Kimura A Ohmichi M Kawagoe J Kyo S Mabuchi S Takahashi T. Induction of hTERT expression and phosphorylation by estrogen *via* akt cascade in human ovarian cancer cell lines. Oncogene (2004) 23(26):4505–15. doi: 10.1038/sj.onc.1207582 15048073

