# Supplementary figures and images for: Relationship between telomere length and the prognosis of breast cancer based on estrogen receptor status: A Mendelian randomization study
Source: Front Oncol. 2022 Oct 21;12:1024772. doi: 10.3389/fonc.2022.1024772 (PMC9634263; doi:10.3389/fonc.2022.1024772)

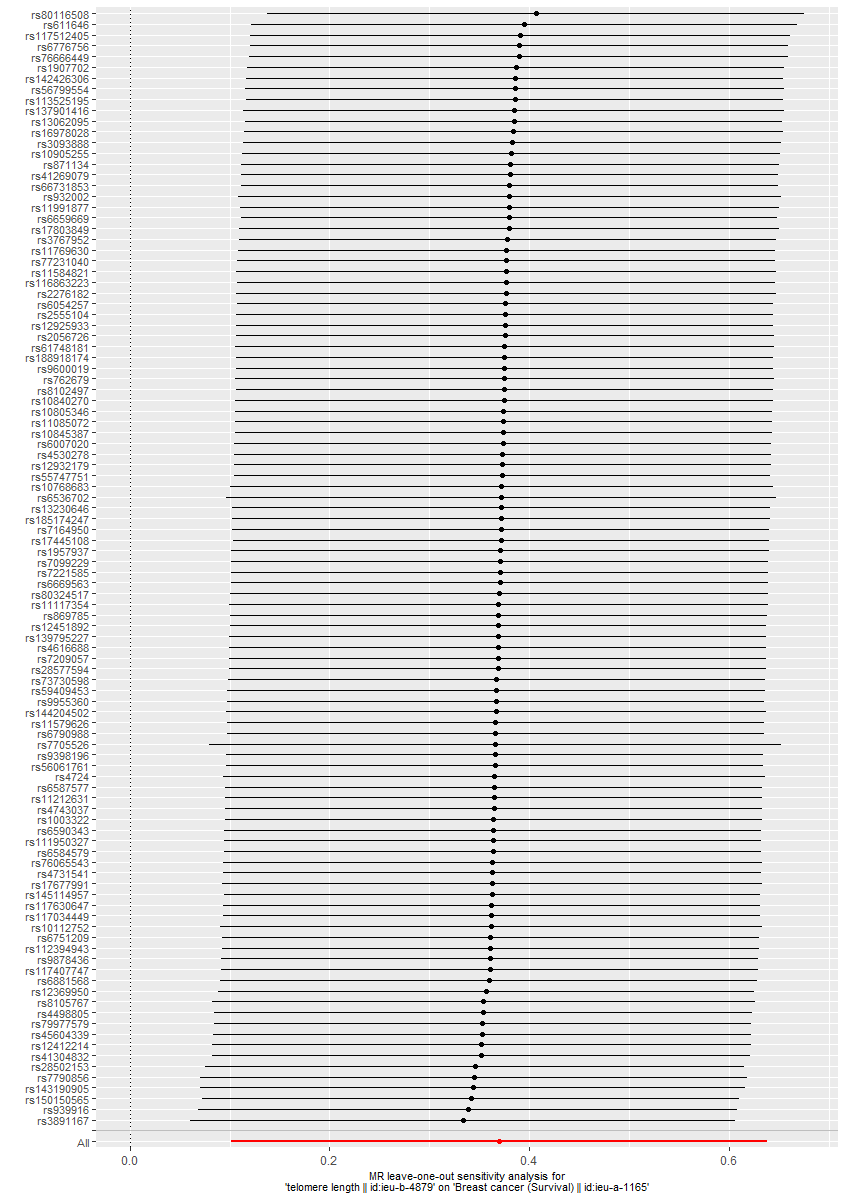

Supplement: Supplementary Figure 1 — Sensitivity analysis based on leave-one-out analysis. [file Image_1.tiff]

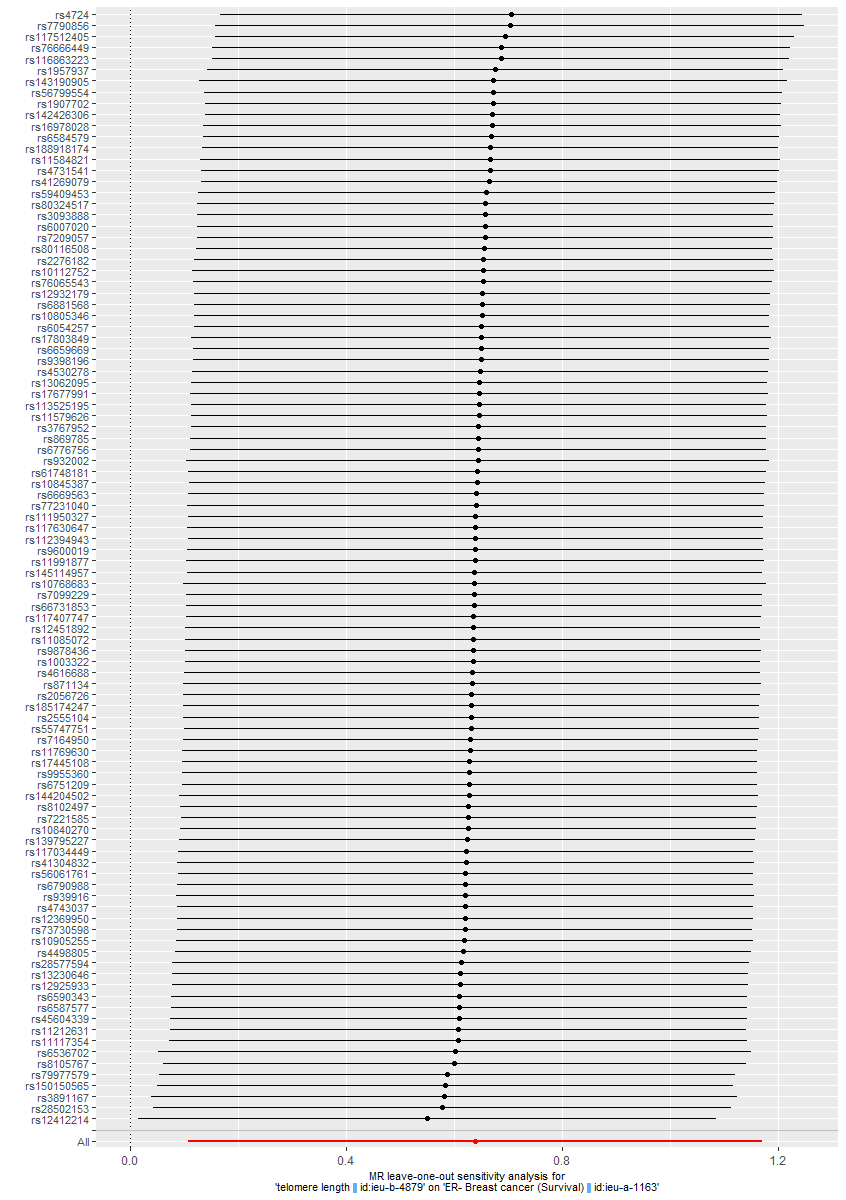

Supplement: Supplementary Figure 2 — Funnel plot to assess the robustness of results. Scattering points represent the effect estimated using a single SNP as an instrumental variable. Vertical lines denote the overall estimate obtained by the inverse variance weighted estimate and the MR-Egger regression. [file Image_2.tiff]

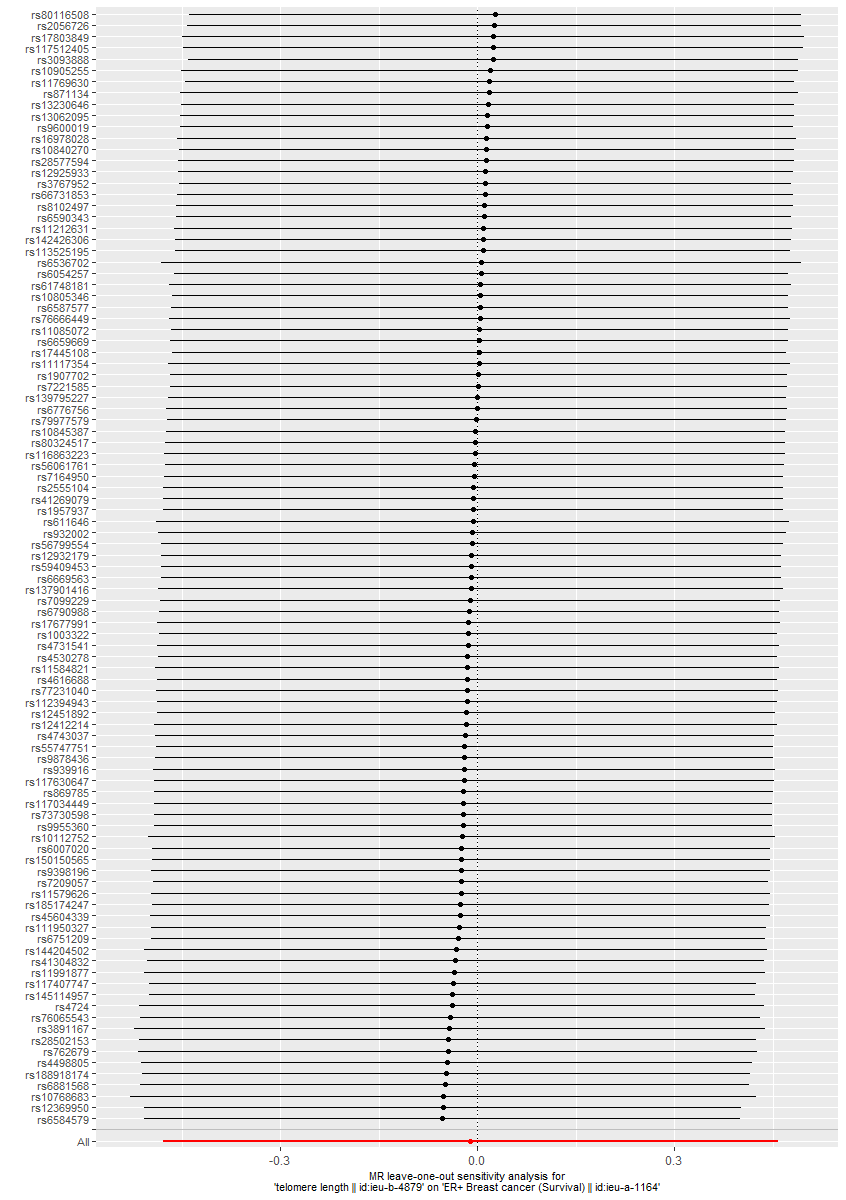

Supplement: Supplementary file 4 [file Image_3.tiff]

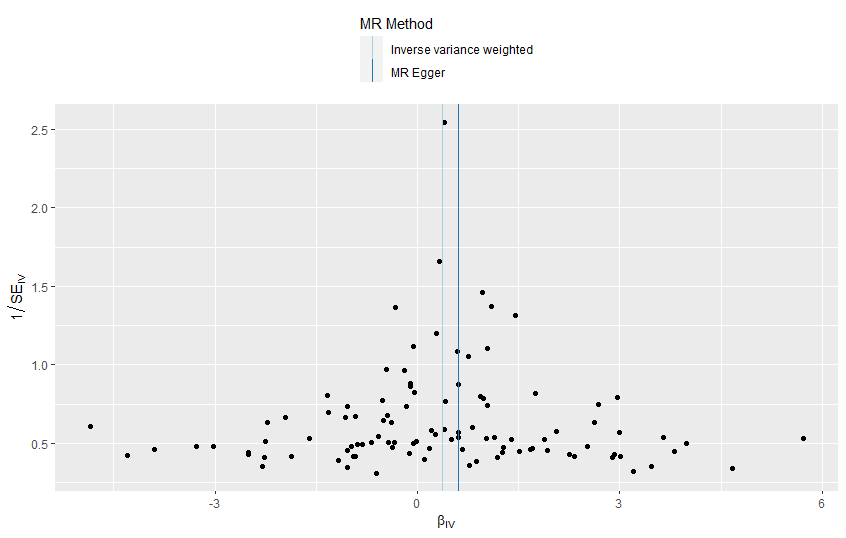

Supplement: Supplementary file 5 [file Image_4.tiff]

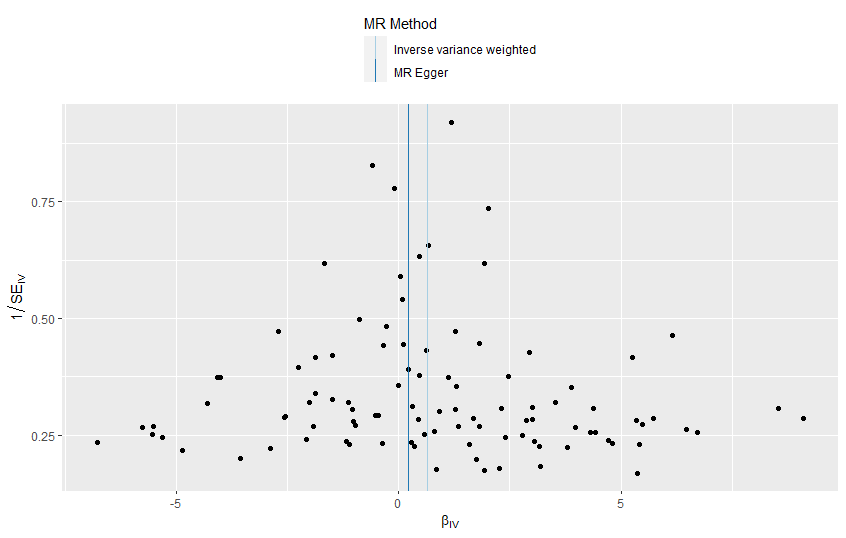

Supplement: Supplementary file 6 [file Image_5.tiff]

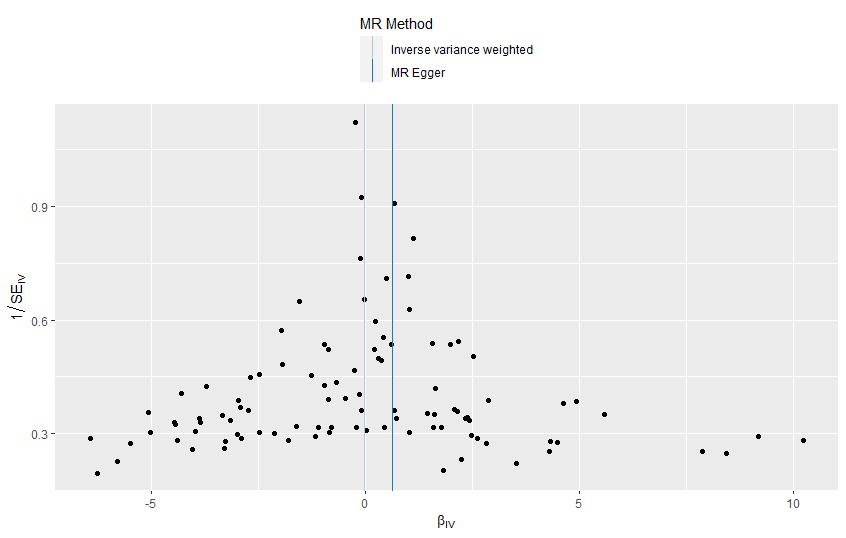

Supplement: Supplementary file 7 [file Image_6.tiff]
